# Strengthening antenatal care providers’ role to address HIV: experiences of pregnant women in Russia

**DOI:** 10.1093/eurpub/ckac131.456

**Published:** 2022-10-25

**Authors:** EJ King, MV Vetrova

**Affiliations:** 1 School of Public Health, University of Michigan - Ann Arbor, Ann Arbor, MI, USA; 2 First Pavlov Medical University, St. Petersburg, Russia

## Abstract

**Background:**

Significant gaps in HIV treatment remain in Russia. Women are often diagnosed during pregnancy, which is important for prevention of mother-to-child transmission and mothers’ longer-term treatment. We aimed to learn the needs of HIV-positive pregnant and postpartum women and to identify opportunities for antenatal care (ANC) providers to better address these needs.

**Methods:**

We conducted in-depth interviews with Russian women who were diagnosed with HIV during pregnancy in July-November 2021. We conducted thematic analysis using team-based deductive and inductive coding of the interview transcripts.

**Results:**

The Russian healthcare system operates in silos, which means that while pregnant women are tested in ANC services, they must visit a specialized AIDS Center for their HIV care. Our respondents mostly appreciated the specialized care, but they recognized that it presented difficulties in discussing their HIV-related needs with ANC providers. Respondents noted a lack of coordination and collaboration between the AIDS Centers and the ANC services. Respondents described how ANC providers were often ill-equipped to answer questions, offer information, or provide extensive counseling upon diagnosis. However, some respondents shared the positive impact a supportive and trustworthy ANC provider had on their engagement in HIV care. This included psychological, informational, and instrumental support. Respondents were receptive to the empathy and openness of ANC providers as long as they did not exhibit incompetence or judgement. The stigma and discrimination that many respondents encountered in the healthcare setting served as significant barriers to openly discussing HIV with ANC providers.

**Conclusions:**

Issues of trust, knowledge, support, compassion, de-stigmatization, and coordination of services are important considerations in strengthening the role that ANC providers can have in improving HIV-positive pregnant women’s engagement in HIV treatment.

**Key messages:**

• Pregnant women who are diagnosed with HIV require more comprehensive psychosocial support from the healthcare system. This could have a positive impact on longer-term engagement in HIV treatment.

• Antenatal care providers need better preparation and empathy to address the concerns of HIV-positive pregnant women and to support these women through the process of accessing HIV treatment and care.

